# Supplementary material for: Freely foraging macaques value information in ambiguous terrains
Source: Sci Rep. 2026 Jan 7;16:881. doi: 10.1038/s41598-025-32879-x (PMC12783851; doi:10.1038/s41598-025-32879-x)
Supplement: Supplementary file 2 — Supplementary Information 1. [file 41598_2025_32879_MOESM2_ESM.pdf]

Supplemental figures

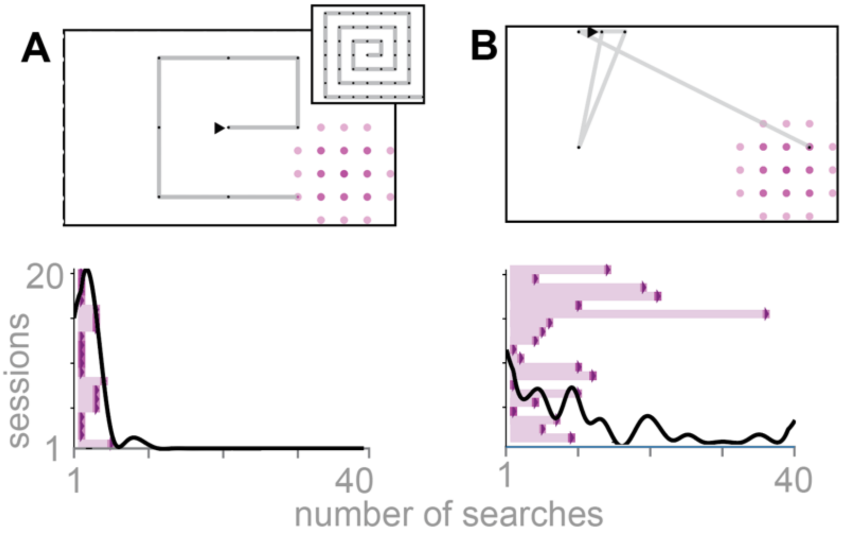

Figure S1 | Simulations of a systematic search algorithm, performed by a hypothetical informed searcher (A) and a purely random searcher (B). A) The informed searcher knows that the one and only reward patch spanned at least 3 piles in each row or column. Therefore, it started from the center of the grid and performed a stripe search, moving on a square spiral path with a stripe width of 3, and sampling every three piles until finding a filled pile. This was to ensure that the informed searcher does not miss the patch while minimizing the number of samples as well as the distance traveled. *Top:* an example localized abundance map and the path of the informed searcher. The black triangle specifies the first search pile. *Bottom:* Raster of foraging outcomes in each simulated foraging session. The probability distributions of the first successful pile search for each monkey (black lines) overlap the raster. **B)** The random searcher chose the next pile on the grid at any distance or angle. *Top and bottom:* same as A.

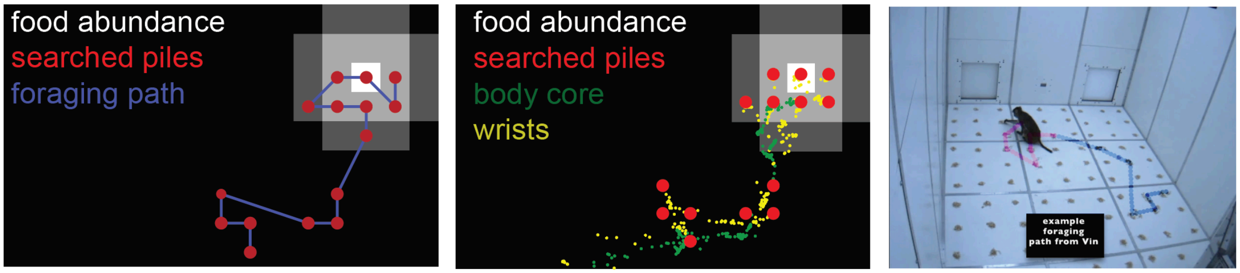

Figure S2 | The foraging and locomotion paths during an example foraging trip. *Left:* the foraging path (blue), searched locations (red), and the hidden localized reward patch (white). *Middle:* the location of the center of the body (green) and the right wrist (yellow). *Right:* the foraging path shows as blue for the subpath before finding the first reward and pink for the subpath after that. The photo is a still image from a movie clip included as Supplementary Video 1.

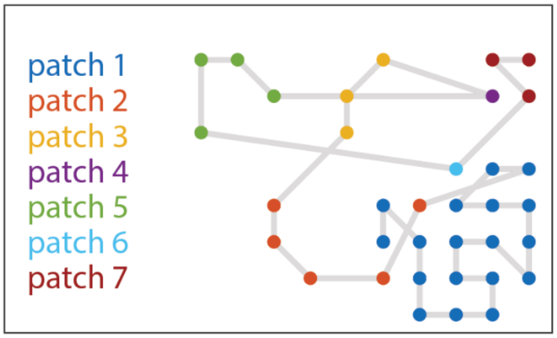

Figure S3 | A foraging session of monkey Hum in which the foraging path resembles a patch-wise search. A patch was identified as a set of consecutive searches for which the step size between each search and the previous search is smaller than 1.5 meters. A patch-wise pattern was not consistently observed across other sessions/monkeys. Instead, we found unimodal distributions of step sizes, as in Fig. 2C.

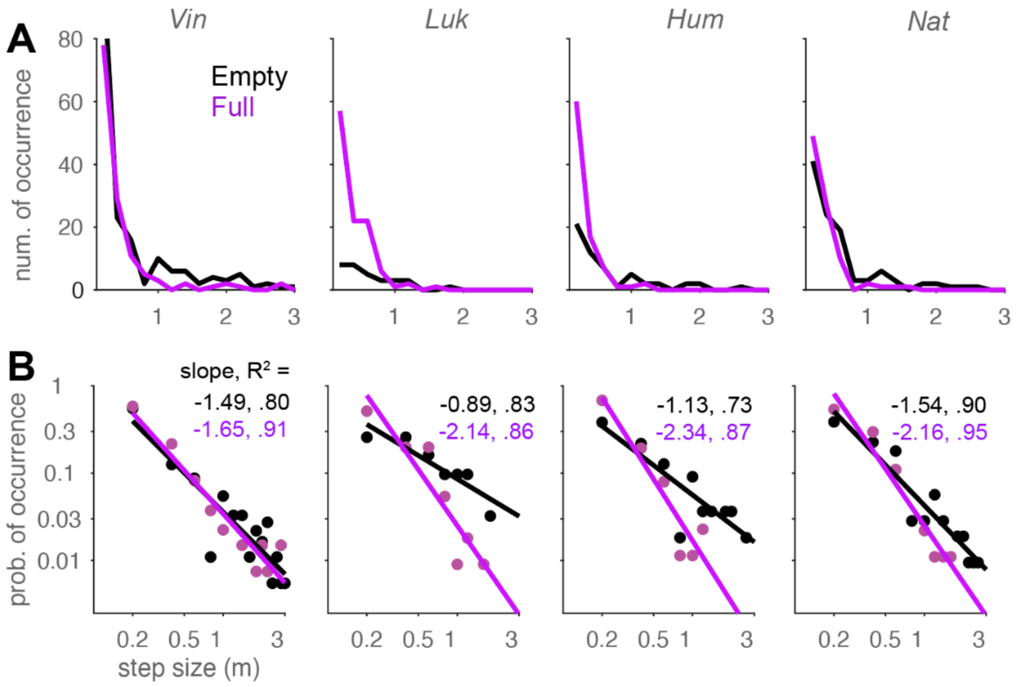

Figure S4 | Step size distributions were close to a Lévy-like random walk. A) Same as Figure 3C, but separated for searches after full and empty piles. **B)** Same as Figure 3D but separated for searches after full and empty piles.

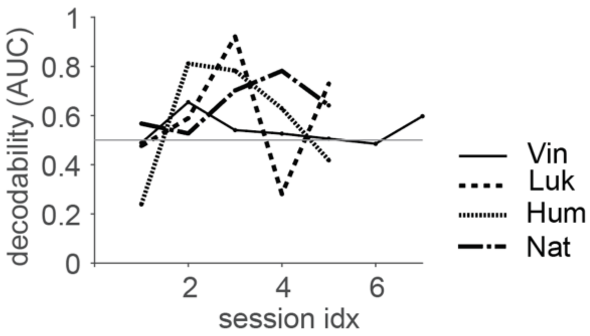

Figure S5 | Decodability of step sizes after encountering filled and empty piles across sessions of one monkey. Decodability was quantified as the area under the R.O.C. The chance level is 0.5. None of the monkeys showed a linear trend across sessions (p = 0.9 (Vin), 0.8 (Luk), 0.9 (Hum), 0.3 (Nat) for a linear regression model).

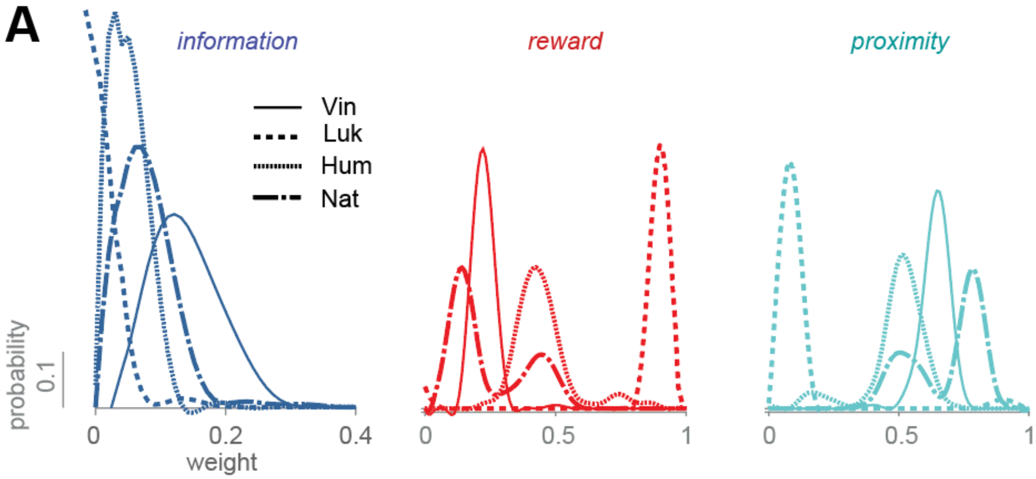

Figure S6 | Comparing weights of the spatial model across monkeys or maps. A) Comparing the information, reward, and proximity weights across monkeys. Vin weighed information higher than other monkeys (decodability > 0.88) while Luk weighed rewards higher than others (decodability > 0.95).

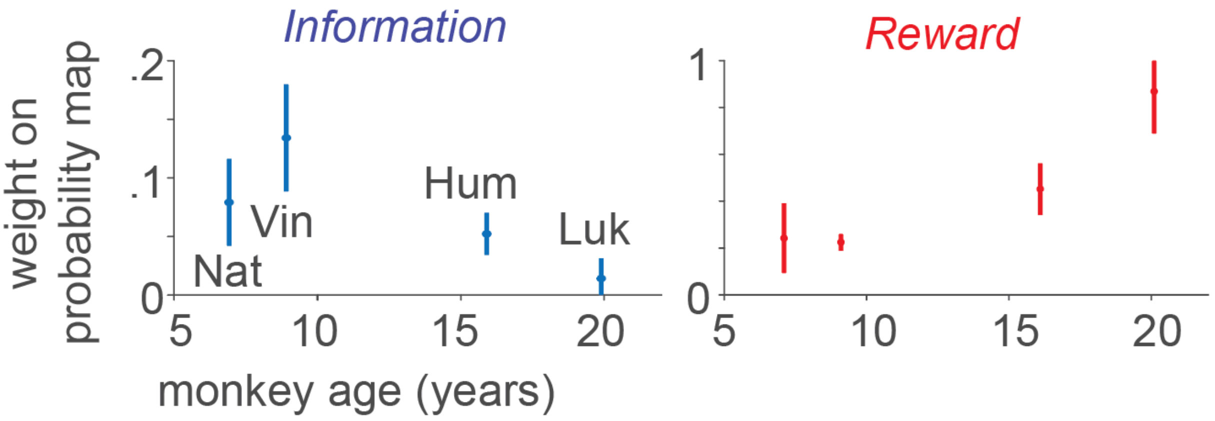

Figure S7 | The information and reward weights as a function of monkeys' age.

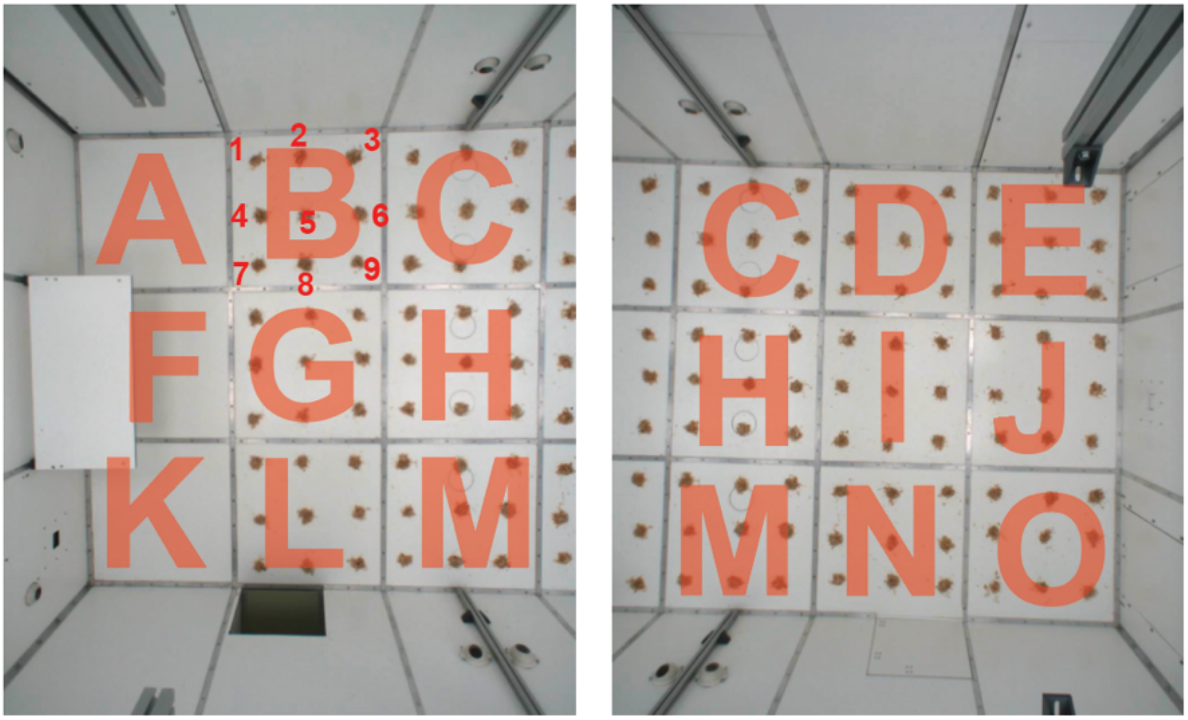

Figure S8 | The bird's eye view of the foraging terrain using two overhead cameras, overlapped with a map of identifying labels for each pile. For example, the topmost left pile was B1, and the bottommost right label was O9.

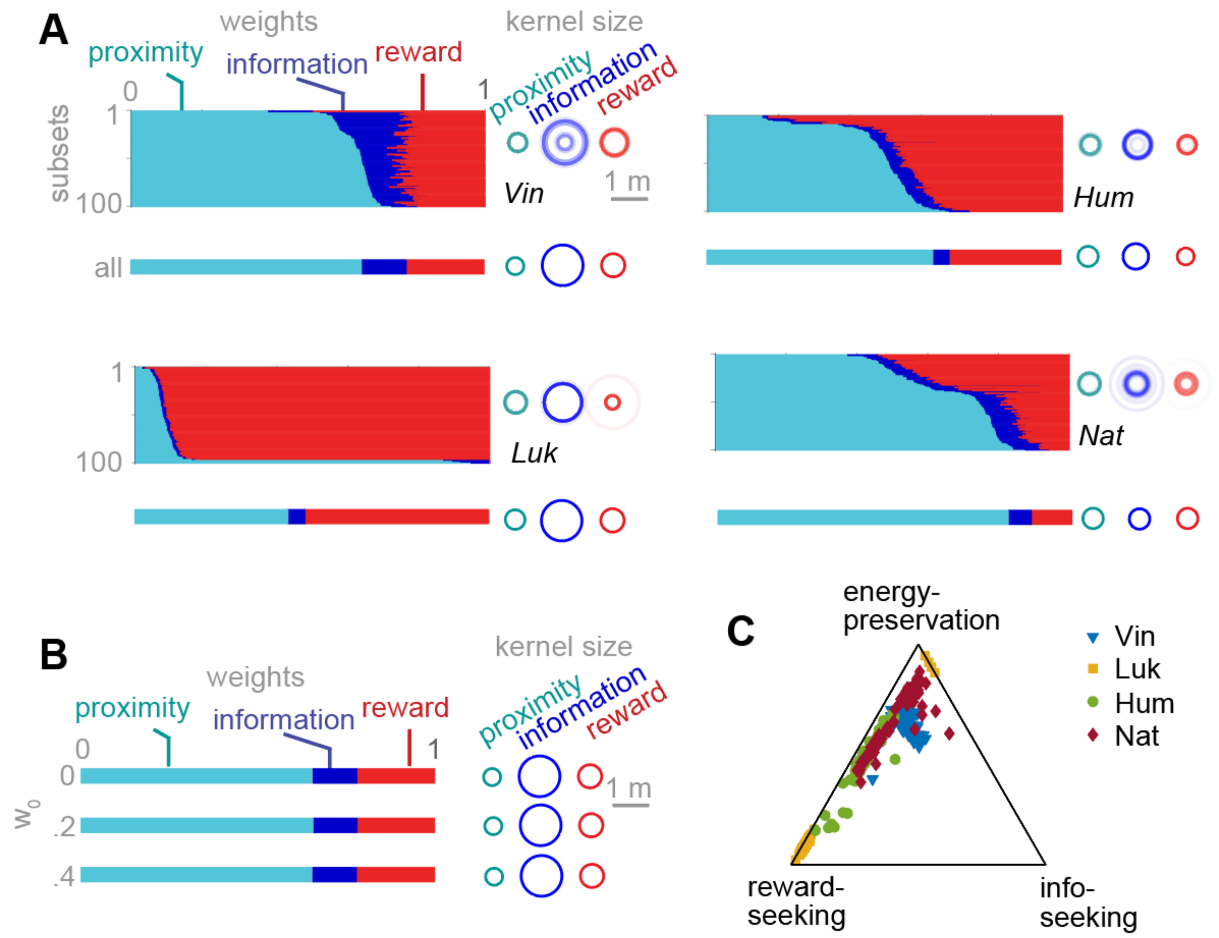

Figure S9 | Additional analyses on fitted model parameters. A *top*) repeated from Fig. 4D. *Bottom*) Estimated model parameters using all of the searches from one monkey. **B)** Estimated model parameters using w_0 as the initial value for w_{info} and w_{rew}. In all other analyses $w_{0,info}$ = $w_{0,rew}$ = 0.3. **C)** A 2D simplex representation of the weights. Each color/marker represents the weights estimated for one subset of searches of one animal.

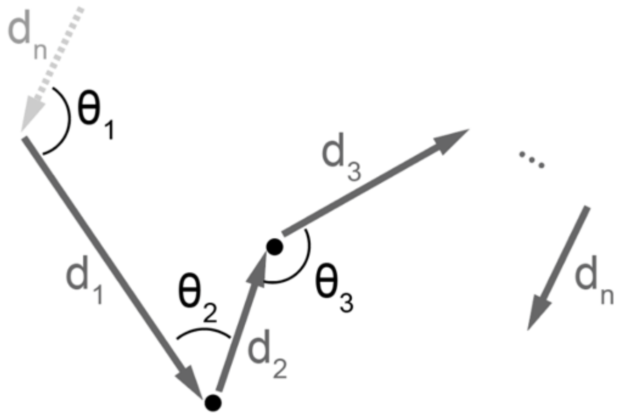

Figure S10 | Method of calculating convolutedness. A hypothetical sub-path, shown as a sequence of steps (vectors) between searched piles (dots). Variables d_i represent the Euclidean distance between consecutive searches, i.e., vector lengths, and variables θ_i represent the angles between each step and the previous one, except for θ_1, for which we use the last step of the path as the step before the first step.

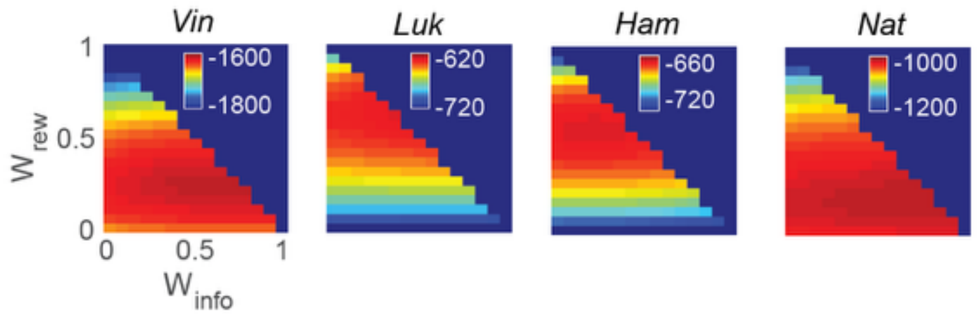

Figure S11 | The log likelihood values in the space of the weights for information and reward maps. The weights were in the range of 0 and 1, with their sum < 1.
